# Research Progress on Sodium Reduction Strategies for Meat Products

**DOI:** 10.3390/foods15172990

**Published:** 2026-08-25

**Authors:** Qian Lu, Peitong Li, Jiangxue Kong, Huijie Li, Fei Shi, Yingchun Zhu, Tengfei Wang

**Affiliations:** College of Food Science and Engineering, Shanxi Agricultural University, Jinzhong 030801, China

**Keywords:** meat products, sodium reduction strategies, saltiness perception, flavor enhancers, salt substitutes, non-thermal processing technologies

## Abstract

Sodium chloride (NaCl) serves multiple functions in meat product processing, including flavor enhancement, preservation, and texture regulation. However, excessive sodium intake significantly increases the risks of chronic diseases, including cardiovascular disease, hypertension, and gastric cancer. Globally, sodium intake among the general population consistently exceeds the daily upper limit recommended by the World Health Organization (less than 2000 mg sodium per day, equivalent to <5 g salt per day). In certain regions, processed meat products are an important source of dietary sodium intake. Consequently, the development of low-sodium meat products has emerged as a critical priority in both the food industry and public health. This review reviews and summarizes the multifunctional roles of sodium chloride in meat products and the underlying mechanisms of these functions, and evaluates mainstream sodium reduction strategies, namely direct sodium reduction, physical modification, salt substitutes, flavor enhancement, odor-induced saltiness enhancement (OISE), and non-thermal processing. The analysis indicates that individual strategies exhibit limitations in terms of sensory quality, safety, or cost. Future efforts should focus on achieving effective sodium reduction through the synergistic application of multiple strategies, without compromising product quality or safety. This review further proposes a product-type-oriented strategy matrix, and multi-strategy synergy combined with AI optimization which is put forward as a promising potential pathway for the industrialization of sodium reduction in meat products, which remains to be validated by further research.

## 1. Introduction

**Figure 1:**
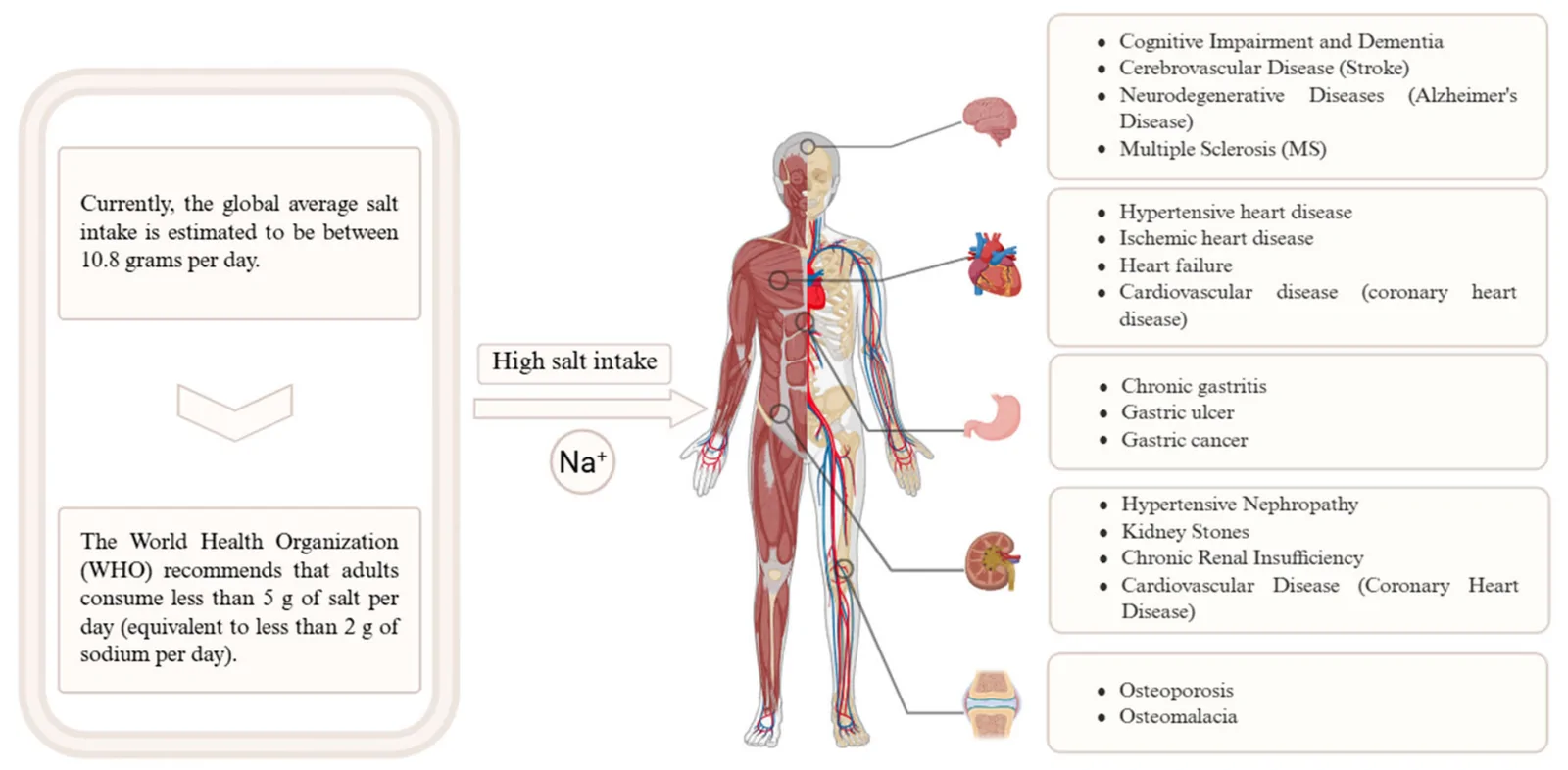
Potential risks to human health from excessive salt intake.

NaCl is an indispensable seasoning in meat processing, providing not only the characteristic salty taste perception, but also exerting multifunctional effects: for preservation, it lowers water activity and inhibits the proliferation of spoilage and pathogenic microorganisms; for texture formation, it promotes myofibrillar protein solubilization and hydration to improve water-holding capacity and gel properties; for flavor development, it modulates endogenous enzyme activities to generate flavor-active substances and balances overall taste profiles [1]. Excessive sodium intake is associated with an increased risk of hypertension and cardiovascular diseases and may also contribute to other adverse health outcomes [2] (Figure 1). Therefore, rational control of sodium intake in food processing and daily diets is essential for maintaining human health. The World Health Organization (WHO) recommends that adults consume less than 5 g of salt per day (equivalent to less than 2 g of sodium per day) [3]. To ensure rigorous conceptual consistency throughout this review, the definitions of related terms are clarified as follows: the term “sodium reduction” specifically refers to the reduction of total sodium content in meat products at the nutritional level; the reduction of added salt/NaCl refers to the adjustment of sodium chloride dosage in actual meat processing. The terms sodium, salt, and NaCl are strictly distinguished throughout the manuscript without ambiguous substitution. Nevertheless, the current global average salt intake is estimated at 10.8 g/d [3], far exceeding the recommended level. Salt intake is in the range of 7–18 g/d across European countries [4], while many Asian countries exhibit high salt intake, with some exceeding 12 g/d [5]. Thus, rational control of sodium intake in food processing and daily diets is of great significance for safeguarding public health.

Raw meat has a low sodium content—for example, beef contains only 63 mg/100 g. After processing, salt addition can reach 4–6% in dry-cured and fermented meat products, whereas most cooked meat products generally contain 1.5–2.5% NaCl [6,7]. Processed meat products are a significant contributor to dietary sodium intake in certain populations [8,9]. Therefore, developing low-sodium meat products has become an inevitable trend.

Existing reviews related to sodium reduction in meat products mainly provide overviews of diverse sodium reduction technical strategies. In contrast, on the one hand, this review employs bibliometric analysis to map out research hotspots and developmental trends within this field, while elaborating on the multifunctional mechanisms of sodium chloride in meat matrices; on the other hand, it attempts to assess available sodium reduction schemes regarding their application potential, cost constraints, and industrial practicability based on existing literature, aiming to offer some references for the practical deployment of salt-reduction work in meat products.

**Figure 2:**
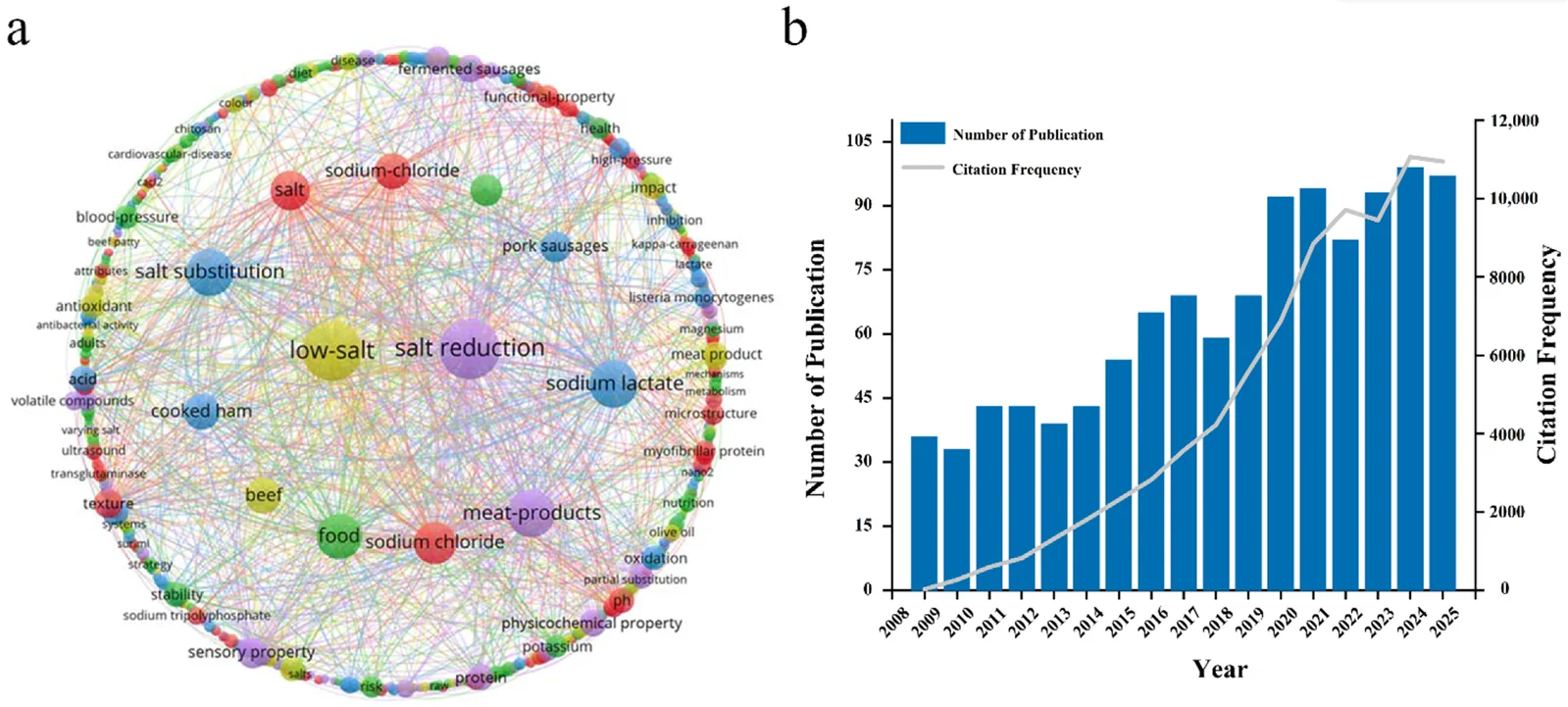
Research trends on meat products with reduced salt. Note: (**a**) Keyword co-occurrence network of research on low-sodium meat products in the Web of Science (WOS) (2009~2025); (**b**) trends in annual publication output and total citation frequency in the Web of Science (WOS).

Bibliometric analysis was performed based on the Web of Science Core Collection database. The retrieval date was 23 January 2025. The time span was set from 2009 to 2025; language was limited to English, and only journal articles were included, excluding reviews, meeting abstracts, editorial materials, and book chapters. The search query was as follows: TS = (sodium reduction OR salt reduction) AND TS = (meat product*). Initial search retrieved 3279 records; duplicates were removed, followed by manual screening of titles and abstracts to exclude irrelevant documents. Finally, 1110 valid records were imported into VOSviewer 1.6.20 for constructing a keyword co-occurrence network (Figure 2a). Origin 2022 was used to plot annual publication number and citation frequency (Figure 2b). For VOSviewer parameters, the minimum number of occurrences of a keyword was set to 5; other parameters were kept as default settings.

Research on low-sodium meat products began in the 1980s and has continued to advance with technological progress. The annual number of publications reflects the development trends of the search topics in the field and, to some extent, reveals the pace of development and the level of research interest [10]. Based on a bibliometric analysis of WOS from 2009 to 2025 (Figure 2), where research hotspots were identified via keyword co-occurrence analysis, the number of publications in this field has steadily increased, while research hotspots have shifted from early-stage “sodium substitution” toward areas such as “saltiness perception” and “novel processing technologies”, reflecting a clear multidisciplinary trend.

To this end, this paper aims to elucidate the multiple functions of sodium chloride in meat products and their underlying mechanisms; comprehensively review and evaluate the technical principles, application efficacy, and limitations of existing sodium reduction strategies; explore the synergistic potential among different strategies and the challenges encountered in industrial applications; and propose potential future research directions and plausible pathways toward the industrialization of sodium reduction technologies from a multidisciplinary perspective.

## 2. Functional Roles and Effects of Sodium Chloride on Meat Products

### 2.1. Functional Roles of Salt in Meat Products

**Figure 3:**
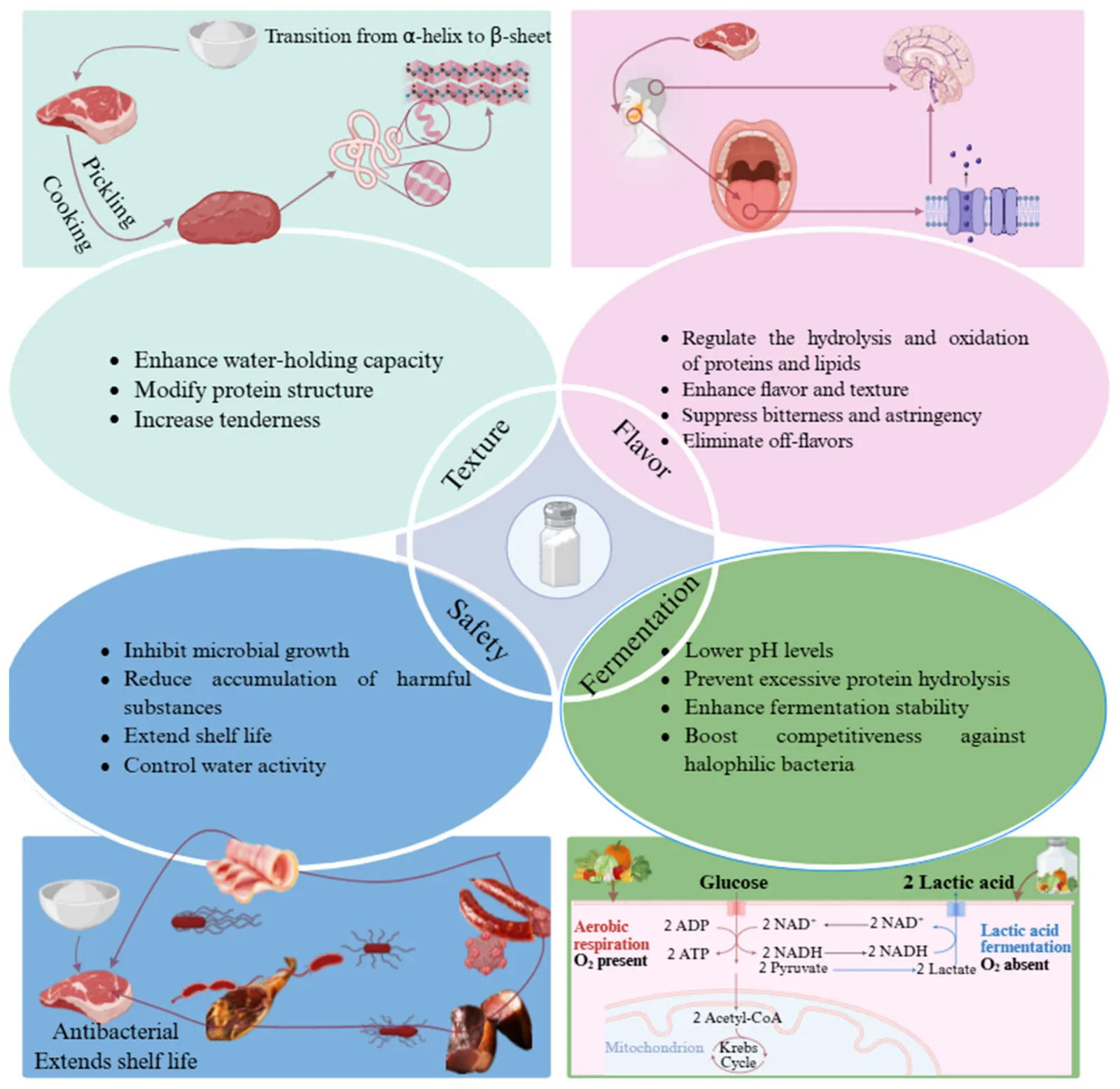
Schematic diagram of the multifunctional roles of NaCl in meat product processing.

In meat processing, sodium chloride functions not only as a salting agent but also as a multifunctional additive. Its roles can be summarized into the following three interrelated aspects (Figure 3): (1) sensory and flavor: serving as the primary contributor to saltiness perception and regulating overall flavor balance through ionic effects [11]; (2) texture and processing: serving as a key determinant of product water-holding capacity, tenderness, emulsifying properties, and viscoelasticity by influencing protein solubility, hydration, and gel network formation; (3) preservation and safety: reducing water activity through osmotic pressure effects while selectively inhibiting the growth of spoilage and pathogenic bacteria. The aforementioned functions arise from the complex interactions between the Na^+^ and Cl^−^ ions dissociated from sodium chloride and the meat matrix (i.e., proteins, lipids, and water).

Notably, meat products contain both major and minor sources of sodium. Although various sodium-based additives (e.g., sodium tripolyphosphate, sodium nitrite, and monosodium glutamate [10]) are incorporated during processing to perform specific functions, sodium chloride typically accounts for more than 80% of the total sodium content, serving as the predominant sodium source [10]. This characteristic indicates that sodium reduction strategies targeting sodium chloride represent the most effective approach for reducing the total sodium content of meat products. By exploring the specific mechanisms of sodium chloride in terms of texture, flavor, and safety, this paper lays a theoretical foundation for understanding the challenges encountered in sodium reduction strategies.

### 2.2. Texture

NaCl is one of the key factors influencing the textural properties of meat products [12]. Its mechanism of action begins with the effects of Na^+^ and Cl^−^ dissociated from NaCl on myofibrillar proteins: the increase in ionic strength promotes the dissolution of myofibrillar proteins, which in turn activates the proteins and alters their spatial conformation. Specifically, Cl^−^ ions bind to positively-charged sites on myofibrillar proteins, increasing the net negative charge of proteins. Na^+^ elevates the ionic strength of the system, screening electrostatic interactions between protein molecules, which weakens protein–protein attraction and promotes protein solubilization, further enhancing protein hydration and water-holding capacity [13]. This, in turn, enhances hydration and water-binding capacity, ultimately improving the binding properties of proteins and the texture of meat products, including water-holding capacity, adsorption capacity, emulsifying properties, and gel stability [14]. The increase in water-holding capacity in meat products reduces cooking loss, thereby enhancing product tenderness and juiciness [13]. Furthermore, the texture and water-retention properties of meat products largely depend on the protein gel formed by salt-soluble myofibrillar proteins (particularly myosin) during thermal processing, which provides cohesion for binding meat pieces [15].

### 2.3. Flavor

The mechanism of salt perception differs from that of sweet, bitter, and umami tastes. While sodium ions dissociated from NaCl constitute the primary source of salty taste in meat products, other cations such as potassium ions can also produce salty sensations, often accompanied by off-tastes including bitterness and metallic notes under certain concentration and matrix conditions. During mastication, Na^+^ diffuses from food to taste receptor cells (TRCs) in the taste buds via the epithelial sodium channel (EnaC) or transient receptor potential vanilloid 1 (TRPV1), thereby eliciting saltiness perception in the taste cortex [16]. The intensity of saltiness perceived by taste receptor cells depends primarily on NaCl concentration; in addition, lipids and proteins in meat products may also contribute to saltiness perception under the action of NaCl [10]. The regulatory effect of NaCl on endogenous lipases and proteases, which facilitates the formation of free fatty acids, amino acids, and salty peptides, is highly dependent on product categories, salt concentration, fermentation conditions, temperature, water activity, and processing duration. Such effects are more pronounced in dry-cured fermented meat products [17,18]. Meanwhile, NaCl can suppress bitterness and boost sweetness, collectively optimizing the overall flavor profile of meat products [19].

### 2.4. Safety

NaCl not only enhances saltiness and flavor but can inhibit the growth of certain spoilage and pathogenic microorganisms (e.g., *Pseudomonas*, *Lactobacillus* spp., *Staphylococcus aureus*, *Listeria monocytogenes*, and *Clostridium botulinum*) in meat products [20]. Its antimicrobial performance is dependent on multiple factors including salt concentration, water activity, product composition, temperature, and microbial species. During the curing process, large amounts of NaCl are added to meat products to impart saltiness, enhance umami, and mask undesirable flavors. By inhibiting the growth of various microorganisms, NaCl influences the flavor and color of meat, thereby extending shelf life and improving product safety. The antimicrobial effect of salt is primarily attributed to its ability to reduce water activity in meat products, and the efficacy of this effect depends on the salt concentration in the aqueous phase of the product. Total added salt cannot fully represent the actual salt concentration in the aqueous phase. Some salt binds to muscle proteins and resides in the bound-water fraction, which cannot generate osmotic stress for microbial inhibition. Only salt dissolved in the free-water-based aqueous phase exerts antibacterial activity. Accordingly, many studies indicate that reduced NaCl levels may shorten the shelf life of meat products, but this effect varies greatly across product types. For example, under vacuum-packaged storage at 5 °C ± 0.5 °C with curing salt (0.6% NaNO_2_) added, a bacon sample pH was in the range of 5.63–5.68 with no inter-group significant differences. Increasing NaCl from 2.3% to 3.5% extended bacon shelf life from 28 d to 56 d [21]. Such salt-dependent shelf life performance is conditioned by product water activity, processing parameters, and storage environment.

## 3. Current Status of Sodium Content in Meat Products and the Rationale for Sodium Reduction

Although fresh meat has a low sodium content—for example, beef contains approximately 63 mg/100 g and pork approximately 70 mg/100 g [11]—processed meat products require the addition of large amounts of sodium chloride during traditional processing to achieve functions such as preservation, flavoring, and texture modulation, resulting in a substantial increase in sodium content. Table 1 presents the typical sodium content ranges of representative meat products, showing that most traditional dry-cured and (non)fermented meat products have a salt content ranging from 2% to 8%, while cooked emulsified meat products generally exhibit lower salt levels.

The World Health Organization recommends a daily sodium intake of less than 2 g (equivalent to approximately 5 g of salt) [30]. Based on a single serving of 100 g, many traditional processed meat products contain high levels of salt, which may easily push consumers’ daily sodium intake above the WHO recommended threshold. Therefore, reducing sodium content in meat products while ensuring product safety and sensory quality has become a critical issue for the food industry and an urgent priority in public health. However, sodium reduction in meat products faces multiple complex challenges: sodium salt serves multiple functions, including flavoring, preservation, and texture modulation; the clean-label trend restricts the use of synthetic additives; and the processing conditions vary significantly across different meat products, necessitating tailored strategies. These factors collectively indicate that sodium reduction in meat products is by no means a simple “subtraction” but rather a systematic endeavor that requires achieving a balance among reducing sodium content, maintaining product functionality and safety, ensuring sensory quality, and adhering to the clean-label trend.

It is also worth noting that regulatory definitions for “low-sodium” and “reduced-sodium” label claims for meat products differ substantially across jurisdictions, which sets different practical targets for product reformulation toward sodium reduction. For commercial pre-packaged processed meat products in China, nutrition content and comparison claims must comply with GB 28050-2011. According to this national standard, the claim of “low-sodium” for pre-packaged solid foods requires sodium ≤120 mg per 100 g, while foods eligible for the “reduced-sodium” claim demand at least 25% sodium reduction compared with reference products. Under U.S. FDA regulation (21 CFR § 101.61), main-dish foods can bear the “low-sodium” label when containing ≤140 mg sodium per 100 g, and the “reduced sodium” claim requires a minimum 25% sodium reduction relative to an appropriate reference food. Such inconsistent regulatory benchmarks should be considered when designing sodium reduction schemes for meat products targeting different markets.

## 4. Advances in Sodium Reduction Strategies

**Figure 4:**
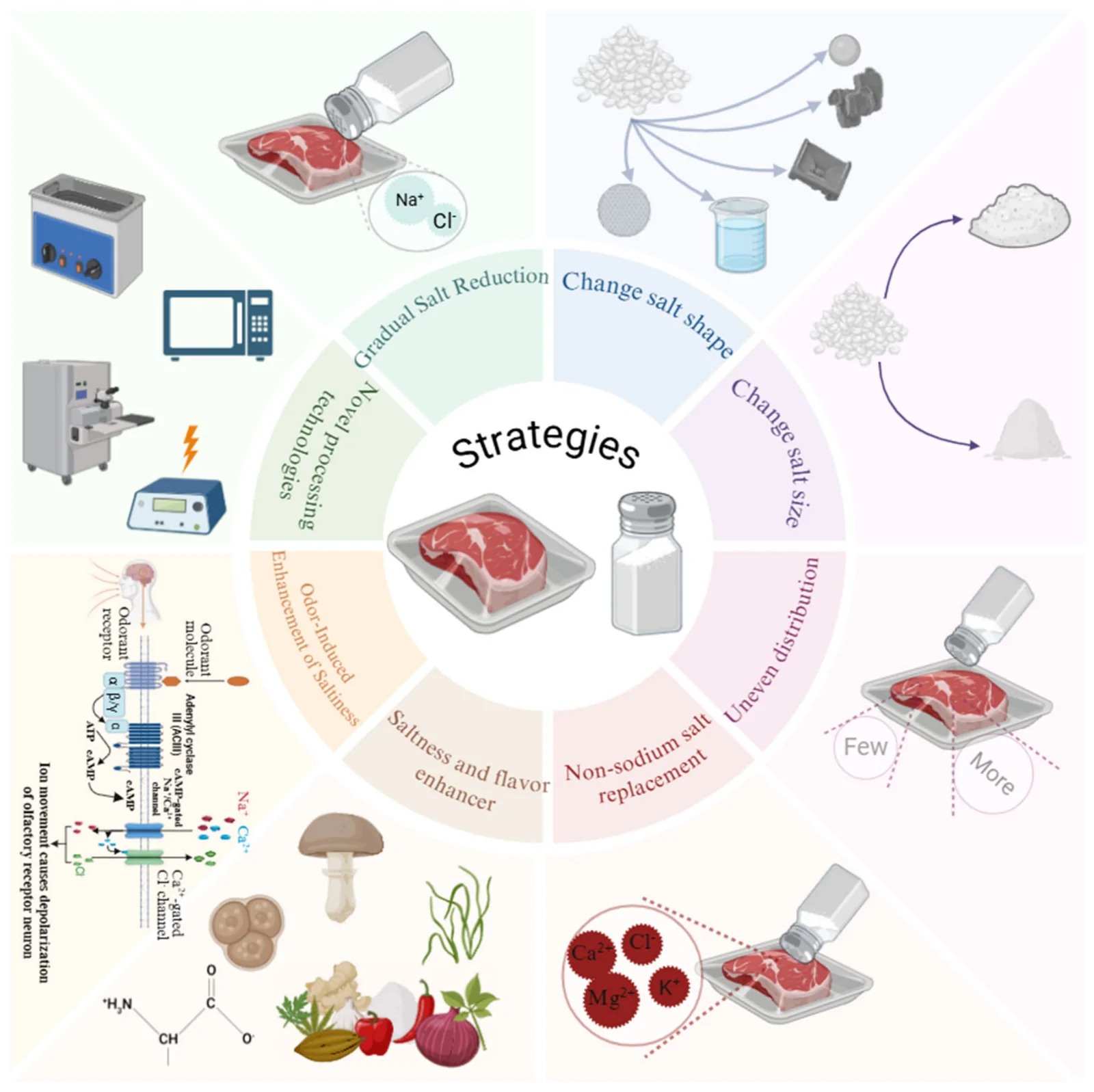
Strategies to reduce salt content in meat products.

The main sodium reduction strategies for meat products focus on the following aspects (Figure 4): (1) directly reducing sodium content; (2) altering the physical form of sodium salt; (3) food matrix structure design; (4) using salt substitutes to completely or partially replace sodium salt; (5) use of flavor enhancers; (6) odor-induced saltiness enhancement; (7) application of novel processing technologies.

### 4.1. Direct Sodium Reduction—Gradual Reduction Strategy

Directly reducing the amount of NaCl added is the most straightforward approach; however, it may lead to flavor deterioration and a shortened shelf life [31]. Therefore, building on direct sodium reduction, adopting a gradual reduction strategy—that is, progressively decreasing the addition level—can prevent noticeable differences in consumer perception [32]; however, such an approach requires industry-wide coordination and entails a long implementation period. For example, when the sodium chloride content in bacon is reduced from 3.5% to 2.3%, sensory properties such as saltiness, juiciness, and texture are adversely affected [33].

### 4.2. Modification of the Physical Form of Sodium Chloride

Altering the physical form of sodium chloride is an important strategy for achieving sodium reduction without compromising product quality. The particle size of salt is closely related to its dissolution rate and diffusion rate [34]. Therefore, modifying the particle size of salt serves as an effective approach for sodium reduction in meat products, including flaked sea salt, nano-sized ultrafine salt particles, hollow salt, and emulsions. The core of this strategy—altering the physical form of sodium chloride—lies in appropriately increasing its specific surface area or forming hollow microspheres, thereby enhancing saltiness perception without compromising food flavor [34].

Micronized salt increases the specific surface area by reducing particle size, thereby improving saltiness perception efficiency, enabling the salty taste to be maintained even with a 50% NaCl reduction, and when combined with ultrasound and KCl, it compensates for texture defects [35]; hollow salt microspheres can achieve a 15% sodium reduction while improving the quality of dough and bread, and this effect has been primarily validated in bread products [36]; micronized fine salt combined with specific salt reduction techniques can achieve a 10–45% reduction in sodium content in blue-veined cheeses, and this effect has been primarily validated in cheese products [37]; emulsified salt delivers NaCl in the aqueous phase of emulsions. Its oral-responsive design enables rapid sodium release, reducing sodium while maintaining saltiness. Though mainly for semi-solid/liquid foods and not yet validated in meat, it is a promising sodium reduction strategy [38].

Sodium chloride with modified physical forms—such as flaked sea salt, nano-sized ultrafine salt particles, hollow salt, and emulsified salt—have demonstrated their sodium reduction benefits across various food applications, achieving significantly enhanced saltiness efficiency without compromising consumer sensory acceptance. However, research in this area remains in its early stages and faces multiple challenges: successful cases of hollow salt prepared by spray drying are relatively limited; the production costs of nano-sized ultrafine salt particles are high; achieving a balance among saltiness intensity, technological properties, and sensory acceptability of flaked sea salt remains difficult; and the molecular mechanisms underlying saltiness perception in emulsion systems remain poorly understood.

Physical modification strategies enhance saltiness perception efficiency by increasing the specific surface area or controlling release, which is potentially compatible with the clean-label trend. Nevertheless, some physical modification technologies may require processing aids, carriers, or emulsifiers during manufacturing, which may compromise the clean-label attribute depending on the formulation; however, they face challenges such as high production costs, limited effectiveness in wet-curing systems, and poor adaptability to existing processing equipment.

### 4.3. Food Matrix Structure Design

Food matrix structure design is an innovative sodium reduction strategy that enhances saltiness perception by modulating the heterogeneous distribution of salt or employing biopolymers to delay Na^+^ release, thereby achieving substantial sodium reduction without compromising sensory quality [19]. This technology shows potential to achieve salt reduction in meat products without compromising saltiness or requiring the addition of sodium substitutes or flavor enhancers, yet practical implementation still faces multiple technical obstacles. For example, in meat products, embedding salt in edible coatings or creating localized high-salt regions can create taste contrast, thereby enhancing saltiness perception [39]. Approaches such as salt coatings [39], fat encapsulation [40], using the coacervates of soy protein isolate and apple pectin, the dispersed coacervate system of soy protein isolate and apple pectin (AP) was treated by high-voltage electrostatic field to achieve inhomogeneous spatial distribution of sodium ions, and this strategy was applied to pre-prepared chicken patties, achieving a 43.59% reduction in sodium content [41].

Food matrix engineering enhances taste contrast by creating heterogeneous salt distribution or delaying ion release, representing a promising pathway for achieving substantial sodium reduction. Its advantages lie in its independence from chemical additives and its substantial sodium reduction efficacy. However, the technical complexity and cost of this strategy constitute the main obstacles: (1) it requires precise processing control, such as multilayer co-extrusion and microencapsulation; (2) biopolymers (e.g., specialty hydrocolloids) are associated with high costs; (3) it may alter the traditional texture of products, thereby affecting consumer acceptance. This strategy is currently more suitable for application in restructured meat products and comminuted meat products, where the matrix is relatively homogeneous and amenable to control; in contrast, achieving heterogeneous salt distribution in whole-muscle products (e.g., steak, ham) poses significant technical challenges.

### 4.4. Partial or Complete Replacement of Sodium Salt with Salt Substitutes

The use of salt substitutes represents one of the most common sodium reduction strategies for reduced-sodium foods. Some salt substitutes can improve product palatability, whereas high-concentration potassium chloride, magnesium chloride, and calcium chloride may instead reduce palatability. Common metal salts include potassium, calcium, and magnesium salts. Although the physicochemical properties of these metal ions are similar to those of Na^+^, exceeding a certain addition level may impart undesirable flavors to the product, such as bitterness, astringency, and metallic notes [42]. In addition, lithium salts exhibit the closest flavor profile to sodium salts; however, due to the toxicity of Li^+^, they are unsuitable as salt substitutes. Although these metal ions each have their limitations, they can exert functionality and antimicrobial effects similar to those of Na^+^ in meat product processing when used appropriately.

Potassium salt, as the most common metal salt substitute, has been widely adopted in meat product processing. Substituting a portion of NaCl with KCl not only inhibits the growth of spoilage microorganisms such as Pseudomonas, Vibrio, and Staphylococcus in meat products but also contributes to maintaining intracellular osmotic pressure, acid–base balance, and normal cardiac function with adequate intake [43]. Studies have shown that substituting 30% of NaCl in pork with 20% KCl and 10% (*w*/*w*, within salt-replacer blends) other ingredients significantly improves gel structure and functional properties [44]. Potassium chloride is the most commonly used substitute. Studies have shown that replacing 50% (*w*/*w*, relative to the original NaCl addition level) of sodium chloride with potassium chloride can maintain protein functionality and ensure the microbiological stability of meat products [45]. Calcium salts (e.g., calcium chloride and calcium lactate) can improve the texture and color of low-sodium meat products; however, excessive addition may lead to textural deterioration. For example, replacing a portion of NaCl with calcium salts in sausages improves product color and texture without compromising hygienic safety [46]. Replacing 25% (*w*/*w*, relative to the original NaCl addition level) of NaCl with magnesium chloride enhances the water-holding capacity and improves the color of pork sausages [47]. Composite substitution using magnesium, potassium, and calcium salts can achieve sodium reduction of 40% (*w*/*w*, relative to the original NaCl addition level) in some products without significantly compromising sensory properties [48]. Therefore, the salt substitution strategy (particularly using KCl) is technologically mature; however, the issue of bitterness limits the substitution ratio, and high potassium content warrants caution. This strategy is applicable to almost all categories of meat products but should be used in combination with flavor masking agents (e.g., amino acids, yeast extracts).

### 4.5. Application of Flavor Enhancers

Human taste perception is typically influenced by a variety of taste-active substances. Flavor enhancers not only compensate for the sensory quality decline induced by certain salt substitutes but also improve the color, texture, and water-holding capacity of products; consequently, they have been widely adopted in reduced-sodium meat products. Currently, commonly used flavor enhancers include umami substances, basic amino acids, salty peptides, and nucleotides [49,50,51,52]. In the development of reduced-sodium meat products, the sole use of salt substitutes tends to result in decreased sensory quality, whereas the synergistic effect of flavor enhancers can compensate for the flavor deficiency resulting from sodium reduction.

Umami substances (e.g., yeast extract and shiitake mushroom extract) enhance saltiness perception and effectively compensate for flavor loss when applied in reduced-sodium fermented sausages and frankfurters [53]. Nevertheless, several commercially available umami enhancers, such as monosodium glutamate and disodium 5′-inosinate, are sodium-containing compounds. Their addition introduces extra sodium into the system, which may offset part of the sodium reduction benefit. Therefore, for low-sodium product formulation, non-sodium-based flavor-enhancing alternatives should be prioritized wherever feasible.

Basic amino acids (e.g., lysine and arginine) inhibit heterocyclic amine formation, improve water-holding capacity, and compensate for sensory defects induced by mineral salt substitutes [54].

In addition, studies have shown that three novel salty dipeptides from bovine bone achieved salt reduction rates of 73.72–77.28% in a sodium chloride solution model system; however, such a high sodium reduction rate was only obtained in sodium chloride solution systems and has not yet been verified in real meat product matrices [55].

Flavor enhancers indirectly enhance saltiness perception by augmenting umami and enriching flavor complexity, serving as an effective strategy to compensate for flavor loss resulting from sodium reduction. Their advantages include a wide range of sources (e.g., natural extracts and fermentation products), relatively controllable costs, and the ability to improve the overall flavor profile. However, excessive use may introduce off-flavors (e.g., the fermented note of yeast extract) or lead to flavor imbalance. From a regulatory and labeling perspective, certain flavor enhancers (e.g., monosodium glutamate) are considered safe but face negative consumer perceptions; meanwhile, “natural” extracts present challenges related to ingredient standardization and supply stability. This strategy is particularly suitable for products with complex flavor profiles and a high demand for umami, such as soups, soy-simmered products, and seasoned meat pieces; in contrast, its application should be exercised with restraint in high-end fresh prepared meat products that aim to highlight the intrinsic flavor of the ingredients.

### 4.6. Odor-Induced Saltiness Enhancement

#### 4.6.1. Basic Principles and Core Pathways of Odor-Induced Saltiness Enhancement (OISE)

Odor-induced saltiness enhancement is a sodium reduction strategy based on cross-modal perceptual interactions. The underlying scientific principle is that when specific aroma compounds are perceived simultaneously with saltiness, the brain integrates the two sensory inputs, leading to a stronger subjective perception of saltiness even though the sodium content remains unchanged [56]. OISE does not alter the direct response of taste receptors but instead achieves synergistic amplification of gustatory and olfactory signals at the level of the central nervous system.

#### 4.6.2. Application of Odor-Induced Saltiness Enhancement (OISE) in Meat Products

In recent years, researchers have identified characteristic aroma compounds with saltiness-enhancing potential from various traditional meat products (Table 2). For example, pyrazines and sulfur-containing compounds are effective saltiness enhancers; 2-furfuryl mercaptan significantly enhanced salty sensation in NaCl + MSG solutions [57]; 2-methoxy-4-vinylphenol in Hunan cured meat significantly enhanced the saltiness of NaCl solutions at concentrations ranging from 1 to 1000 μg/L [58]. These findings suggest that the complex flavor substances inherent in traditional fermented meat products may serve as a natural reservoir for OISE. It is worth noting that the enhancement effect of OISE is concentration dependent. Multiple studies have shown that under low-sodium conditions (e.g., 0.2%~0.5% NaCl solution) [59], the aroma-induced saltiness enhancement effect is more pronounced. This implies that the OISE strategy is particularly suitable for synergistic application with sodium reduction goals—that is, compensating for the loss of saltiness perception through characteristic aromas in products with already reduced sodium content.

#### 4.6.3. Influence and Constraints of the Meat Product Matrix on the Efficacy of Odor-Induced Saltiness Enhancement (OISE)

Translation of OISE from solution systems to meat products faces several matrix-specific constraints. First, the high fat and protein content of meat matrices can adsorb and bind aroma compounds, reducing their effective concentration during oral processing [58]. Second, many salt-enhancing volatiles (e.g., pyrazines, furanthiols) are susceptible to degradation or loss during thermal processing and storage [63]. Third, the aroma profile of traditional fermented meat sausages is highly dependent on formulation and processing conditions, leading to considerable batch-to-batch variability and making it difficult to consistently achieve the desired sensory characteristics [64].

#### 4.6.4. Synergistic Potential of Odor-Induced Saltiness Enhancement (OISE) with Other Sodium Reduction Strategies

OISE can potentially complement other sodium reduction strategies, but the level of empirical support varies considerably. Empirically supported strategies include the combination with KCl substitution—characteristic meaty and smoky aromas can mask potassium-associated bitterness while enhancing perceived saltiness [65]. Theoretical proposals lacking meat-product validation are as follows: (1) coupling OISE with physically modified salts (e.g., hollow salt) to achieve temporal complementarity of rapid Na^+^ release and sustained aroma-driven enhancement; (2) microencapsulation of salt-enhancing aromas within structured food matrices to program sequential release during mastication. These latter schemes remain conceptual and require dedicated validation in meat systems [10].

#### 4.6.5. Industrial Prospects and Limitations of Odor-Induced Saltiness Enhancement (OISE) Strategies

Overall, OISE is an innovative, clean-label-compatible strategy, but its industrial application in meat products is still in its infancy. Key bottlenecks include aroma stability during processing, matrix interference, and the high cost of natural aroma compounds. Currently, OISE is most plausibly suited as an auxiliary strategy for low-temperature or short-time heated ready-to-eat meat snacks, applied synergistically with salt substitution or physical modification rather than as a standalone solution [66].

### 4.7. Novel Processing Technologies

Sodium reduction in meat products can be realized not only by lowering NaCl addi-tion, but also by improving the functional properties of meat matrices to support low-sodium formulations [67]. Current novel processing technologies include high-pressure processing (HPP), ultrasound (US), pulsed electric fields (PEF), microwave technology, and electrodialysis. These technologies disrupt cell membrane structures to boost the diffusion and permeation of salt-related compounds. They improve saltiness perception, water-holding capacity, and tenderness retention under low-NaCl conditions, which makes lower-sodium formulations practically feasible, rather than directly removing sodium from meat products [68].

#### 4.7.1. High-Pressure Processing (HPP)

HPP is a non-thermal processing technology widely applied for developing low-sodium meat formulations. It cannot reduce the intrinsic sodium content of meat products directly; instead, it improves the solubility of myofibrillar proteins under low-sodium conditions, optimizes gel structure, and enhances perceived saltiness, enabling meat products to maintain acceptable quality at lower NaCl addition levels. In low-salt emulsified sausages, high-pressure processing at 200 MPa can impart textural and sensory characteristics that are more preferred by consumers [69]. For example, treatment at 300 MPa increases the content of umami nucleotides [70]; high-pressure treatment at 100 MPa for 5 min maintains the water-holding capacity, texture, and sensory quality of the product [71].

#### 4.7.2. Ultrasound Technology

US disrupts muscle fiber structures through cavitation and facilitates salt penetration, and 26.8 kHz has exhibited favorable performance under specific experimental conditions. This technology significantly improves pickling efficiency and water-holding capacity. For example, treatment with 26.8 kHz ultrasound significantly improves the water-holding capacity of pork, disrupts myofibrillar structure, and accelerates the pickling process [72]; treatment with 20 kHz ultrasound for 120 min significantly enhances salt permeability, water-holding capacity, and tenderness of spiced beef, while promoting the expansion of muscle tissue by disrupting muscle fiber structure, thereby improving overall product quality [73].

#### 4.7.3. Pulsed Electric Field (PEF) Technology

PEF is a non-thermal technology that enhances mass transfer through electroporation. McDonnell [74] demonstrated that PEF treatment significantly accelerates salt uptake during the salting of pork, indicating its effectiveness in improving diffusion kinetics in meat products; at a field strength of 0.52 kV/cm, the sodium content of beef jerky was significantly reduced by 34%, with sensory scores comparable to those of the control group and no adverse effects on oxidative stability or microbial safety [25].

#### 4.7.4. Microwave Technology

Microwave technology utilizes electromagnetic radiation at frequencies ranging from 300 MHz to 300 GHz and enhances saltiness perception by modulating protein aggregation and water distribution. Microwave heating modifies the microstructure of muscle-based foods, improving salt diffusion and saltiness perception by modulating protein aggregation and water distribution [75]. A 915 MHz microwave treatment combined with starch processing (heating for 4 min followed by holding for 5 min) significantly improves the gel properties of low-sodium silver carp surimi, increasing the water-holding capacity (WHC) to 0.91 [76]. Microwave-assisted thermal sterilization combined with the addition of herbs reduces the salt content of chicken pasta meals by 50% while maintaining saltiness perception intensity comparable to that of the control group [77].

#### 4.7.5. Other Novel Processing Technologies

Vacuum curing technology effectively shortens curing time and promotes the uniform distribution of NaCl in meat products. Vacuum impregnation increases the contact area between the brine solution and muscle tissue, thereby improving mass transfer efficiency and shortening the curing time of meat products. Vacuum tumbling technology utilizes physical impact to induce collision, friction, and compression of muscle-based foods, thereby effectively enhancing the quality of cured meat products. For example, Barat [78] reported that replacing traditional pile salting with simultaneous brine thawing–salting reduced the total curing time of Spanish ham by 61% compared to conventional methods. Gamma irradiation does not enhance the saltiness of low-sodium meat products; however, it effectively inhibits the growth of Listeria monocytogenes and other microorganisms. Therefore, the primary application value of gamma irradiation lies in replacing a portion of sodium salt in high-salt products to achieve shelf-life extension. Studies have shown that gamma irradiation effectively extends the shelf life of low-sodium hot dog sausages, with an irradiation dose of 1.5 kGy significantly reducing the microbial load while neither significantly affecting sensory acceptability nor causing excessive lipid oxidation [79].

In summary, novel processing technologies offer new approaches for addressing texture and shelf-life challenges associated with sodium reduction by physically modifying muscle structure, enhancing mass transfer, or improving protein functional properties. Their advantage lies in their ability to “empower” low-salt systems, typically without introducing chemical residues. However, the high equipment investment and operating costs represent the primary bottlenecks for industrialization. In addition, minor fluctuations in process parameters can significantly affect product quality, necessitating stringent process control. Energy efficiency is also a key consideration, with the energy efficiency ratio of certain technologies (e.g., ultrasound) still requiring improvement. These technologies are better suited for high-value-added products with stringent requirements for texture and freshness, such as ready-to-eat low-temperature poultry products and high-end hams; they can also serve as pretreatment steps in combination with traditional processes.

## 5. Future Perspectives on Sodium Reduction in Meat Products

Reducing the sodium content in meat products is a complex and systematic endeavor that requires achieving an optimal balance under multiple constraints, including sensory quality, microbial safety, processing feasibility, cost control, and regulatory compliance. Although various sodium reduction strategies have shown potential at the laboratory scale, their industrial application still faces significant challenges (Table 3). Future breakthroughs will depend on multi-strategy synergistic innovation, product-type-specific precise design, and the integrated application of emerging technologies.

### 5.1. Multi-Strategy Synergy and Intelligent Design

A single sodium reduction strategy often fails to balance multiple objectives. For example, direct reduction or salt substitution may compromise product texture and shelf life, whereas over-reliance on flavor enhancers may run counter to the clean-label trend. Therefore, multi-strategy synergy is regarded as a promising direction for future development [35]. The application of intelligent optimization tools, particularly artificial intelligence (AI) and machine learning (ML), is expected to potentially bring about a paradigm shift in the R&D process for reduced-sodium products, though relevant practical applications still require further exploration. Predictive models use existing literature and experimental data to train models that predict the comprehensive effects of different salt substitute combinations, flavor-enhancer types, and processing parameters on the sensory attributes, texture, and shelf life of the final product. Reverse formulation design first defines the target objectives for the product (e.g., “sodium content reduced by 30%, hardness deviation from the control ≤ 10%, no bitterness”), then employs AI algorithms to search for and recommend optimal ingredient combinations and processing conditions, thereby significantly shortening the R&D cycle. Sensomics data integration correlates instrumental analysis data (e.g., from electronic tongue, electronic nose, and texture analyzer) with large-scale consumer sensory evaluation data to construct a quantifiable “composition–structure–perception” quality map, thereby enabling precise regulation of products.

Nevertheless, translating these physical field technologies from laboratory-scale trials to full-scale manufacturing still faces tangible practical barriers, such as the complexity of equipment operation, difficulty in precise parameter optimization, and inconsistent effects on product quality and uniformity during continuous industrial production [81]. Lab-optimized multi-strategy formulas and processing parameters frequently cannot be directly reproduced under large-scale production conditions due to mass-transfer heterogeneity and batch-to-batch raw-material variation [82]. Additional bottlenecks include inconsistent global regulatory thresholds for low-sodium nutritional claims, safety assessment requirements for novel salt-replacing components, and the scarcity of high-quality, standardized multi-dimensional datasets for robust AI-model training [83,84]. Consequently, multi-strategy synergy combined with AI optimization remains a prospective technical pathway rather than a turn-key industrial solution, and real-world validation under manufacturing conditions is indispensable.

### 5.2. Product-Type-Specific Sodium Reduction Pathways

Different meat products exhibit significant variations in processing technologies, texture foundations, water activity, and flavor formation pathways; therefore, sodium reduction strategies must be appropriately tailored to each product type (Table 4).

### 5.3. Technology Transfer and Industrialization Bottlenecks

Many strategies that succeed at the laboratory scale may encounter significant challenges during large-scale production. Key challenges for industrialization include cost issues: the cost of nano-sized ultrafine salt particles, specific salty peptides, and natural flavor enhancers (e.g., high-purity yeast extract) being substantially higher than that of ordinary table salt; high equipment investment: introducing HPP, PEF, and other equipment entails substantial capital expenditure and high energy consumption; poor process stability: the addition of emulsified salt and microencapsulated salt demands extremely high precision in mixing uniformity and temperature control, making batch-to-batch variability likely during scale-up; standards and regulations: novel salt substitutes, engineered nanomaterials, and new processing aids require lengthy safety assessments and regulatory approval. It should be noted that nano-sized salt particles and microencapsulated salt are conceptually different from engineered nanomaterials, with distinct safety considerations and regulatory requirements. Inconsistent standards for “low-sodium” and “sodium-reduced” across different countries and regions pose challenges for product labeling and international trade.

### 5.4. Regulations, Consumer Perception, and Sustainability

Aligning with clean-label and regulatory compliance trends, future sodium reduction strategies should be compatible with the “clean-label” concept, prioritizing naturally sourced ingredients and clearly stating their functions; employing dynamic sensory analysis methods (e.g., temporal dominance of sensations and temporal check-all-that-apply) to elucidate the dynamic flavor profile of reduced-sodium products during mastication, combined with consumer segmentation studies to enhance product acceptance; incorporating sustainability assessment to systematically evaluate the full lifecycle impact of sodium reduction strategies, for example, whether the mining of potassium salts places pressure on the environment; whether non-thermal processing technologies genuinely reduce overall energy consumption; whether novel ingredient sources (e.g., seaweed) are sustainable.

The future of sodium reduction in meat products will be a systematic process driven by deep multidisciplinary integration and whole-industry-chain collaboration. This requires food scientists to devise more sophisticated formulations, engineers to develop more efficient equipment, sensory scientists to gain insights into more nuanced consumer demands, and policymakers to foster a more innovation-friendly environment. Only through such systematic efforts can we effectively address the public health challenges posed by excessive sodium intake while continuing to enjoy the palatability of meat products.

## 6. Conclusions

Sodium chloride remains indispensable for the sensory, textural, and safety attributes of meat products, yet the public health imperative to lower dietary sodium intake compels the industry to pursue effective reformulation strategies. This review confirms that no single sodium reduction approach can simultaneously satisfy all requirements for saltiness, texture, shelf life, cost, and consumer acceptability. From a practical standpoint, salt substitution (especially KCl) and flavor enhancers (e.g., yeast extracts, amino acids) are technologically mature, relatively low cost, and already widely deployable in industrial production, although off-taste masking and regulatory compliance regarding potassium labeling remain considerations. Conversely, physically modified salts, matrix structure design, and novel processing technologies have demonstrated clear sodium reduction potential in laboratory studies, but their translation to commercial scale is constrained by high capital investment, process instability, and batch-to-batch variability, placing them at the experimental-to-pilot stage for most meat applications. OISE is conceptually attractive and compatible with the clean-label trend, yet its industrial application remains nascent due to aroma stability, matrix interference, and high costs of natural active compounds; it is best positioned as an auxiliary strategy rather than a standalone solution.

Looking forward, the industrial viability of low-sodium meat products will not depend on a single technological breakthrough, but rather on the synergistic integration of multiple strategies tailored to specific product matrices, ideally guided by AI-assisted formulation optimization, supported by harmonized regulatory frameworks and informed by dynamic sensory evaluation of consumer perception. Specialized combination schemes should be adopted for dry-cured fermented meat, emulsified sausages, restructured meat, and ready-to-eat meat snacks according to their distinct processing characteristics. This multi-pronged, product-oriented approach represents the most feasible pathway towards achieving meaningful and sustainable sodium reduction without compromising the quality and safety of meat products.

## Figures and Tables

**Table 1:** Representative processed meat products and their salt content.

No.	Product Types	Representative Product	Processing Technology	Native Salt Content/%	Reduced-Salt Treatment/%	Sodium Reduction Research Findings	Evaluation Method
1	Dry-cured non-fermented meat	Bacon [22]	Cured, soaked in brine, then smoked or air-dried	4	2.68	Replacing 33% NaCl with 22% KCl and 11% calcium ascorbate significantly enhanced aroma, color, and overall acceptability, while enriching volatile flavor compounds	Sensory acceptability evaluation
2		Salted meat [23]	Fresh bellies were dry-salted with the salt mixtures, refrigerated for 48 h, washed, dried for 60 h, heated twice at 90 °C for 1 h with a 48 h rest between, kept at 10 °C/30–35% RH for 1 day, sliced, vacuum-packed, and stored at 4 °C for up to 60 days.	4	1.8	Both alternative salting methods reduced sodium by one-third to one-half (from 1.6 g Na/100 g to 0.75–1.07 g), ensured microbiological stability during storage, and BGW showed higher 2-methylbutanal content, while FFA levels were not significantly affected	Physicochemical analysis
3		Smoked bacon [24]	Bacon was cured in brine at 10 °C for 4 d, immersed in 5% smoke aroma solution for 180 min, roasted at 5 °C for 48 h, vacuum-packaged, and ripened at 0 °C for 14 d	4	1.2~2.8	70% KCl substitution significantly enhanced proteolysis and protein oxidation, while lipid oxidation remained unaffected, and protein oxidation was positively correlated with proteolysis	Physicochemical analysis
4		Beef jerky [25]	Prepared by cutting beef into strips, marinating, and then drying or roasting	2	1.2	Reducing salt content to 1.2% combined with pulsed electric field (PEF) treatment resulted in no significant differences in flavor, saltiness, or overall acceptability compared to the control group	Consumer acceptability test
5		Jinhua ham [26]	Hams were dry-salted with either 6% or 8% NaCl in multiple additions over 7 days at 0–4 °C and 80–90% RH, and exudates were collected at days 1, 2, and 7	8	6	Low salt led to higher cathepsin B and L activities in exudates and lower release rates, which resulted in higher residues in ham tissues	Physicochemical analysis
6	Fermented meat products	fermented dry sausage [27]	Pork minced, mixed with additives and starter; culture, stuffed into casings, air-dried at 25 °C/30–50% RH for 1 d, then fermented at 2 °C/75–80% RH for 8 d	2.5	1~2	NaCl reduction increased moisture, aw, LAB counts and carbohydrate-derived volatiles, while 2.0% NaCl gave the highest overall acceptability, thus being recommended as the desired level	Physicochemical analysis
7		Chinese sausage [28]	Stuffed into casings after marination, then naturally air-dried in a ventilated, dry environment	6	4.2	A formulation consisting of 18% potassium lactate, 12% lysine, and 70% NaCl serves as an effective alternative to 100% NaCl, reducing sodium content while contributing to flavor quality development	Volatile flavor compound analysis
8		Sour meat [29]	Pork cubes were mixed with salt and rice flour, packed layer-by-layer in sealed jars, and fermented at 25 °C for 28 days	6	3	Low salt resulted in lower pH, higher TTA, higher abundance of Lactobacillus and Lactococcus, higher levels of TFAAs, organic acids, esters, and better sensory scores, with the S3F10 group recommended	Physicochemical analysis

**Table 2:** Summary of saltiness enhancement caused by odor.

No.	Food Matrix	Characteristic Aroma Compounds	Source	Test-System NaCl Concentration/%	Sensory Evaluation Method	Main Results
1	Solid system [57]	4 pyrazines, 5 acids, 4 sulfur-containing compounds, 3 others	Jinhua ham	0.2	Static and dynamic sensory methods	Pyrazines and sulfur-containing compounds are effective saltiness enhancers; 2-furfuryl mercaptan significantly enhanced salty sensation in NaCl + MSG solutions
2	Solid system [59]	(E)-2-Decenal, dodecanal, tetradecanal, decanal, 1-octen-3-ol, benzaldehyde, 2-heptenal, heptanal	Spicy stewed duck	0, 0.2, 0.5, 0.8	Orthonasal-gustatory, retronasal-gustatory, orthonasal-retronasal-gustatory	All eight compounds significantly enhanced saltiness (especially at 0.2% and 0.5% NaCl), with the retronasal pathway producing stronger enhancement than the orthonasal
3	Solid system [60]	1-octen-3-ol, nonanal, heptanal, 2-methylbutanal, 3-methyl-butanal, benzaldehyde, octanal, and 2,6-dimethylpyrazine	Chinese dry-cured hams	0.3	Quantitative descriptive analysis	These eight odorants significantly intensified saltiness perception when added to the low-concentration NaCl solution (0.3%)
4	Solid system [61]	Sardine odor	Sardine	0, 0.06, 0.12, 0.23	Combined taste-olfactory evaluation under cross-modal interaction	OISE occurred only for the sardine aroma and only at low (0.06%) and medium (0.12%) salt concentrations, with no significant enhancement at the highest concentration (0.23%).
5	Solid system [62]	Heptanal, octanal, hexanal, pentanal, 2-pentylfuran	Nanjing water-boiled salted duck	0, 0.25, 0.5, 0.75	Retronasal dynamic sensory evaluation	Five aroma compounds confirmed with saltiness-enhancing effects; heptanal and octanal showed strongest enhancement
6	Solid system [60]	1-octen-3-ol, nonanal, heptanal, 2-methylbutanal, 3-methylbutanal, benzaldehyde, octanal, 2,6-dimethylpyrazine	Chinese dry-cured hams	0, 0.3, 0.75, 0.8	Quantitative Descriptive Analysis	All eight compounds significantly enhanced saltiness at the low 0.3% NaCl concentration, 1-octen-3-ol showing the strongest effect
7	Solid system [58]	2-methoxy-4-vinylphenol had the strongest effect, followed by diallyl sulfide, 2,5-dimethylthiophene, 2,6-dimethylphenol, 2,5-dimethylpyrazine, and 2,3-butanedione	Hunan Larou (smoke-cured bacon)	0.3%	Odorant–NaCl mixture model experiments were used to confirm the saltiness-enhancement ability of the aroma compounds	with sulfur-containing, nitrogen-containing and phenolic odorants showing better enhancement, 2-methoxy-4-vinylphenol demonstrating the strongest enhancing effect

Note: This table summarizes published findings related to OISE. The column “Test-system NaCl concentration” only lists the background NaCl concentration of each test system. The saltiness-enhancing effects and conclusions are described in the “Main Results” column and can be obtained from the corresponding original references.

**Table 3:** Comparative analysis of sodium reduction strategies for meat products.

Strategy	Sodium Reduction Potential	Cost Impact	Industrial Feasibility	Main Sensory Impact	Safety Considerations	Most Suitable Product Types	Least Suitable/Cautiously Recommended Product Types
Direct/gradual reduction [6,31]	Low (≤15%)	Very low	Very high	Reduced saltiness, bland flavor	Increased microbial risk	All types (as a baseline)	Non-sterilized products with high shelf-life requirements
Physically modified salt [10,34]	Medium (20~30%)	High	Medium	Saltiness perception comparable, slight textural changes	Limited additional risks, subject to processing-aid regulatory compliance	Surface-treated products, dry-mixed sausages	Liquid-cured, injection products
Salt substitutes [43,48,80]	Medium to high (25~50%)	Low to medium	High	Possible bitterness/metallic taste	Risk of hyperkalemia (requires labeling)	Widely applicable, especially low-to-medium-grade emulsified products	Poultry, aquatic products with mild flavor
Flavor enhancers [49,50]	Low to medium (10~25%)	Medium	High	Enhanced umami and overall flavor, possible off-flavors	Some may have allergenic potential or regulatory restrictions	Products with complex flavor profiles, soy sauce-braised products, soups	High-end fresh meats emphasizing intrinsic flavor
Odor-induced saltiness enhancement [57,66]	Low to medium (10~30%)	High	Low	Enhanced saltiness perception, altered aroma characteristics	Depends on source of aroma compounds	Low-temperature emulsified products, snack meat products	Products subjected to prolonged high-temperature processing
Food matrix structure design [34,41]	High (30~60%)	High	Low	Altered texture, changes in flavor release profile	Depends on the hydrocolloids/polymers used	Restructured meats, comminuted meat products, plant-based composite products	Traditional whole-muscle products
Novel processing technologies [67,69]	Medium (20~40%)	Very high	Medium to low	Improved tenderness and juiciness, minimal impact on flavor	No chemical residues are generated; safety is conditional on well-controlled processing parameters	High-value-added low-temperature meat products, aquatic products	Most low-cost meat products (cost-limited)

Note: Sodium reduction potential represents the maximum sodium reduction range without substantial loss of sensory acceptability; cost impact is classified according to the percentage increase in total production cost compared with the conventional formula; industrial feasibility is comprehensively evaluated from technical maturity, equipment compatibility and process controllability. Partial interval grading in this table is the comprehensive summary of multiple published studies [6,10]. Specific reduction range, cost and practical constraints for each strategy are supported by corresponding references marked in each cell.

**Table 4:** Sodium reduction strategies for different meat products.

Product Category	Key Challenges	Preferred Strategies	Strategies to Avoid/Use with Caution
Dry-cured/fermented products [20,85] (e.g., ham, sausage)	Long processing time; relies on salt for dehydration, preservation, and flavor regulation	Odor-induced saltiness enhancement (OISE) as core: screen and reinforce salt-enhancing aroma compounds generated during fermentation; partial KCl substitution: use in combination with amino acids, with careful control of substitution ratio; physically modified salt: promote penetration and shorten curing time	Avoid substantial direct salt reduction to prevent spoilage microorganism growth and safety risks
Emulsified products [69,86] (e.g., frankfurter sausages, luncheon meat)	Extraction of salt-soluble proteins and formation of gel networks are critical for texture	Physically modified salt/emulsified salt: enhance protein extraction efficiency; formulated food hydrocolloids (e.g., carrageenan, konjac gum): compensate for gel strength; non-thermal processing technologies: improve protein functionality under low-salt conditions	Avoid high proportions of calcium/magnesium salts that may destabilize the emulsion
Restructured/cured products [87,88] (e.g., steak, cured meat)	Need to ensure tenderness, juiciness, and uniform flavor distribution	Ultrasound/vacuum tumbling: promote penetration of low-concentration brine; flavor-enhancer injection: directly infuse umami substances and salty peptides into the matrix; edible salt coating: create localized high-salt areas on the surface to generate taste contrast	Avoid excessive use of water-retaining agents such as phosphates to align with clean label trends
Ready-to-eat snack products [89,90] (e.g., dried meat slices, jerky)	Low water activity; concentrated flavor; direct saltiness perception	Formulated flavor enhancers: enhance overall flavor; surface spraying technology: precisely distribute salt and aroma compounds on the product surface	Avoid overly complex or artificial flavor profiles

## Data Availability

No newly-generated experimental data are included in this review. The bibliometric supporting data are provided in the Supplementary Materials.

## Supplementary Materials

The following supporting information can be downloaded at: https://www.mdpi.com/article/10.3390/foods15172990/s1.

## Abbreviations

The following abbreviations are used in this manuscript:

NaCl	Sodium Chloride
CaCl_2_	Calcium Chloride
WHO	World Health Organization
OISE	Odor-Induced Saltiness Enhancement
WOS	Web of Science
TRCs	Taste Receptor Cells
EnaC	Epithelial Sodium Channel
TRPV1	Transient Receptor Potential Vanilloid 1
KCl	Potassium chloride
HPP	High-Pressure Processing
US	Ultrasound
PEF	Pulsed Electric Fields
